# Risky business: males choose more receptive adults over safer subadults in a cannibalistic spider

**DOI:** 10.1093/beheco/arac023

**Published:** 2022-04-25

**Authors:** Lenka Sentenská, Catherine Scott, Pierick Mouginot, Maydianne C B Andrade

**Affiliations:** Department of Biological Sciences, University of Toronto Scarborough, 1265 Military Trail, Toronto, Ontario, Canada; Department of General and Systematic Zoology, University of Greifswald, Loitzer Strasse 26, 17489 Greifswald, Germany; Department of Biological Sciences, University of Toronto Scarborough, 1265 Military Trail, Toronto, Ontario, Canada; PSL Université Paris: EPHE-UPVD-CNRS, USR 3278 CRIOBE, Université de Perpignan, 52 Avenue Paul Alduy, CEDEX 9, 66860 Perpignan, France; Department of Biological Sciences, University of Toronto Scarborough, 1265 Military Trail, Toronto, Ontario, Canada

**Keywords:** brown widow spider, immature females, *Latrodectus geometricus*, male mate choice, sexual cannibalism

## Abstract

Understanding factors affecting male mate choice can be important for tracking the dynamics of sexual selection in nature. Male brown widow spiders (*Latrodectus geometricus*) mate with adult as well as immature (subadult) females. Mating with adults involves costly courtship with a repertoire of signaling behaviors, and typically ends with cannibalism (“self-sacrifice” initiated by male somersault). Mating with subadults involves brief courtship with behavioral repertoire reduced to one component (vibration) and no cannibalism. We examined male mate choice as a function of risks associated with different types of mates and the cues available to courting males. Previous studies showed male preference for adults based on air-borne pheromones, but it was unclear whether that preference is maintained after males reach female’s webs. We show that males prefer adults also based on silk-borne contact cues. To determine which types of cues trigger different courtship components, we swapped adults and subadults between webs. We showed that contact with adult females’ webs triggers two courtship behaviors from the repertoire, with adult female’s bodies triggering additional behaviors. However, vibrational signals occur regardless of the web origin or female developmental stage. We conclude that males recognize subadult females as potential mates, but are more likely to invest in costly courtship behaviors and mating attempts with adults. In our experiments, subadults were less likely to mate than adults. We conclude that mating with adults could be the preferred option for males because of the higher likelihood of copulation, even at the cost of a higher risk of cannibalism.

## Teaser

In the brown widow spider, males can mate with non-cannibalistic immature (subadult) females, but they prefer mating with cannibalistic adults, using a more extensive repertoire of courtship behaviours and signals. We show that costly male courtship behaviours are triggered by cues on the female’s web or body. Males engage in web-based courtship behaviours only upon contact with adult webs and costly female-oriented behaviour only upon contact with an adult female. We conclude that the reduced courtship and lack of cannibalism with subadults arises from male choice to reduce investment into subadult mating.

## Introduction

Sexual selection is expected to favor male mate choice when female quality varies and when mating requires high investment from males such as nuptial gifts, lengthy courtship, or fights with rivals (Bonduriansky 2001). Males can exhibit mate choice through the decision of whether to mate or not (precopulatory choice) or through investment into a given mating (post-copulatory or cryptic choice; Edward and Chapman 2011). Male choice may be linked to female traits that predict phenotypic and/or genetic quality (e.g. Weiss and Dubin 2018), fecundity/fertility (e.g. Schlupp 2018), or, in polygamous mating systems, the risk and intensity of sperm competition (Parker 1979; Simmons 2001). Additionally, when mate choice requires forgoing one potential mate in favor of a future mate, factors that are likely to decrease the probability of reaching that future mate can also lead to decreased choosiness (e.g. predation risk, Forsgren 1992; Jennions and Petrie 1997; Edomwande and Barbosa 2020; energetic costs of movement, Milinski and Bakker 1992). Finally, variation in male mating patterns may also arise from variation in the conspicuousness or detectability of different types of females. For example, at one extreme, juvenile females may not produce sexual signals at all (e.g., Gaskett 2007; Bilen et al. 2013). In this case, the search for evolutionary determinants of male mate choice is unlikely to be fruitful (Castellano et al. 2004).

Spiders are interesting subjects for studies of male mate choice because they are typically protandrous, and in many species, the first mate has substantial paternity benefits (first male sperm priority; Elgar 1998; Huber 2005, but see e.g. Tuni et al. 2020). First male priority also makes pre-copulatory choice costly for males, as forgoing one mate may decrease the likelihood of being the first to copulate. In some species, mating with certain females incurs a higher risk of cannibalism and the loss of potential future reproduction (Elgar 1992). If such females are detectable, selection could favor increased male choosiness to avoid cannibalism (Johnson et al. 2011; MacLeod and Andrade 2014; Baruffaldi and Andrade 2015). For example, mating with molting or freshly molted females eliminates the risk of cannibalism since these females are unable to attack their mates because they cannot move while their exoskeleton is still soft (Lubin 1986; Uhl et al. 2015). This tactic is common in spiders (Jackson 1986a; Danielson-François et al. 2012; Gregorič et al. 2016), and may lead to males seeking out immature females.

Although males of most spider species that cohabit with immature females mate with them during or shortly after molting, in three species of widow spiders (genus *Latrodectus*) mating occurs a few days before the molt to maturity with females late in their subadult instar (i.e. “immature mating”; Biaggio et al. 2016; Baruffaldi and Andrade 2020; Sentenská et al. 2020). After maturation, females mated as subadults produce similar (*L. hasselti* and *L. geometricus*; Biaggio et al. 2016; Baruffaldi and Andrade 2017) or even greater numbers of offspring compared with adult-mated females (*L. hesperus*; Baruffaldi and Andrade 2020). When paired with adult females, widow spider males engage in lengthy courtship on the web and the female’s body (Ross and Smith 1979; Segoli et al. 2008; Harari et al. 2009; Scott et al. 2015), whereas the courtship towards late-subadult females is significantly reduced (Biaggio et al. 2016; Baruffaldi and Andrade 2017; Sentenská et al. 2020). Additionally, in two widow spider species, *Latrodectus hasselti* and *L. geometricus,* males mating with adult females perform a copulatory “somersault” which triggers female cannibalism, limiting males to a single mating (Forster 1992; Andrade 1996; Segoli et al. 2008). In both self-sacrificial species, however, males mating with subadults typically survive mating, as they only rarely somersault (*L. hasselti*) or somersaults are reported to be absent (*L. geometricus*; Biaggio et al. 2016).

Additionally, males of widow spiders break off parts of their paired copulatory organs inside the female paired genitalia (Snow et al. 2006; Segoli et al. 2008; Andrade and MacLeod 2015) that may block insemination by rivals and serve as mating plugs. The loss of these parts does not prevent male widow spiders from inseminating subsequent females (Andrade and MacLeod 2015), in contrast to the genitalic mating plugs of many other spiders (e.g. Fromhage and Schneider 2005). Mating plugs were found within both spermathecae at a higher frequency in subadult-mated females compared with adult-mated females (Sentenská et al. 2021), which may contribute to increased paternity for males that mate subadults.

Spider males generally use chemical cues and signals to search for and choose between their mates. Males initially follow air-borne pheromones to localize females (although they may also follow the silk of other males, Scott et al. 2019), then respond to chemical and tactile cues to initiate courtship (Gaskett 2007). Finding a subadult female and mating with her seems to be highly beneficial for males due to the apparently reduced courtship effort required and the increased likelihood of surviving the mating. When given a choice, however, males of the brown widow spider *L. geometricus* released at a distance from females on their webs approach adults more frequently than subadults (Waner et al. 2018; Sentenská et al. 2020). Similarly, widow spider males were shown to be more attracted to the presence, silk or chemical extracts of unmated adult females over those of subadult females (e.g. Anava and Lubin 1993; Stoltz et al. 2007; Waner et al. 2018; Sentenská et al. 2020). This may indicate that males either actively prefer adult females or that subadult females produce a weaker chemical signal than adults or no signal at all, in which case male choice is “passive” and does not necessarily indicate a preference for adults (e.g. Castellano et al. 2004). In the absence of adult females, however, both early- and late-subadult females of *L. geometricus* on their webs were readily approached by males from a distance, although only late-subadults were courted after the male reached their web (Sentenská et al. 2020). This indicates that males recognize subadult females as mating partners from a distance and are able to determine whether they are ready to mate upon contact with their webs or body odor/cues. Contact pheromones on the female’s silk or body may be key to this discrimination as such cues provide details about a female’s quality as a mate because they generally contain more precise information than air-borne pheromones (Tietjen and Rovner 1982; Gaskett 2007; Thomas 2011; Uhl 2013, Baruffaldi et al. 2019). Previous studies explored only choices based on air-borne (i.e. less specific) cues (Waner et al. 2018; Sentenská et al. 2020), therefore it is not yet clear whether males of *L. geometricus* would prefer adult over late-subadult females if exposed to contact pheromones of both females simultaneously.

Male widow spiders typically court adult females using a repertoire of vibrational signals (Sivalinghem and Mason 2021) and behaviors (Ross and Smith 1979; Segoli et al. 2008; Harari et al. 2009; Scott et al. 2015). Previous studies showed that males perform reduced courtship in webs of late-subadult females (Biaggio et al. 2016; Baruffaldi and Andrade 2017; Sentenská et al. 2020) and no courtship in webs of early-subadult females (Sentenská et al. 2020), suggesting that males acquire stage-specific information upon entering a female’s web. However, from these studies, it is not possible to discern whether this information is gained solely from the female’s web or whether the female’s presence in the web affects male behavior as well. Key courtship components for *L. geometricus* include courtship on the web, at a distance from the female (“distal” courtship, Scott et al. 2015), such as vibrational signal production and web reduction behavior (adding silk and cutting and bundling sections of female’s web). Contact courtship components (“proximal” courtship, Scott et al. 2015) include standing on the female’s abdomen and laying silk strands on her body (“mate binding”; Ross and Smith 1979; Segoli et al. 2008; Harari et al. 2009; Scott et al. 2015). Although vibrational signals are produced by males courting females of both stages, web reduction and mate binding is typical only for courtship towards adults and has rarely been observed during courtship towards subadult females (Biaggio et al. 2016; Baruffaldi and Andrade 2017; Sentenská et al. 2020). It is not known what exactly triggers the different courtship components and whether different cues (contact with web vs. contact with the female’s body) may trigger different behaviors. Additionally, males apparently do not perform somersaults when mating with subadult females (Biaggio et al. 2016) and it is unclear whether males adopt this behavior only after contact with the female herself or whether contact with an adult female’s web is sufficient.

Here we conducted two studies to investigate male mate choice and courtship behavior in the brown widow spider *L. geometricus*. In the first study, we tested the prediction of a preference for subadults based on contact, silk-borne pheromones in a two-choice Y-maze experiment in which males were exposed to silk of adult and late-subadult females simultaneously. In the second study, we designed an experiment to disentangle whether contact with silk or the female’s body, or both, drives differences in the timing and occurrence of courtship behavior and mating with subadult and adult females. We ran a “web swap experiment” in which we placed adult and subadult females in the webs of other females. This created four scenarios exposing males to different combinations of cues (adult female in adult web, subadult female in adult web, adult female in subadult web, and subadult female in subadult web). We predicted that differences in distal courtship (on the web before contact with the female) would be driven by silk cues, whereas later stages of courtship (proximal, in contact with the female’s body) and copulation would be driven by cues associated with the female (body and/or behavior).

## METHODS

### General protocol

Thirty adult females were collected in Los Angeles, California, USA (34.11023591671581, −118.29157616275369) in January 2020, from spatially separated webs. Females were transported to the laboratory at the University of Toronto Scarborough, placed into plastic containers (5 × 5 × 7 cm), and fed weekly with a cricket (*Acheta domesticus*). When females produced an egg sac, it was removed from the female’s container and placed into a larger container (9 × 9 × 11.5 cm). After hatching, spiderlings were kept together and fed twice a week with *Drosophila* sp. fruit flies. After the second molt, individual spiderlings were transferred into separate containers and fed twice weekly with fruit flies. Males were fed fruit flies throughout their life and females were fed one cricket per week from approximately the fifth instar until they became adults. All individuals were reared under laboratory conditions, in a temperature-controlled room at 25 °C (12:12 h light:dark cycle).

### Study 1: male mate choice based on female silk

We tested the effect of female developmental stage (late-subadult vs. adult) on male mate choice based on contact with silk. The readiness to mate occurs in late-subadult females and is marked by color change of her genital area from pale gray to dark brown; typically 4–6 days before the final molt (Biaggio et al. 2016; Sentenská et al. 2020). Subadult females (*n* = 40) were used for the experiment two to three days after this change was observed and were checked for molting after the experiments. To control for age, adult females (*n* = 40) were used in trials 5–13 days after the molt to maturity and adult males (*n* = 40) were used 6–20 days after the molt to maturity. Ten subadult females did not molt to maturity within 6 days after the experiments (indicating that they were not ready to mate at the time of the experiment), so the trials using their silk were not included in the analyses (final *n* = 30 trials with subadult females). Males were never exposed to silk produced by females from their own family lines. Individuals of both sexes were used only once in the study.

**Figure 1:**
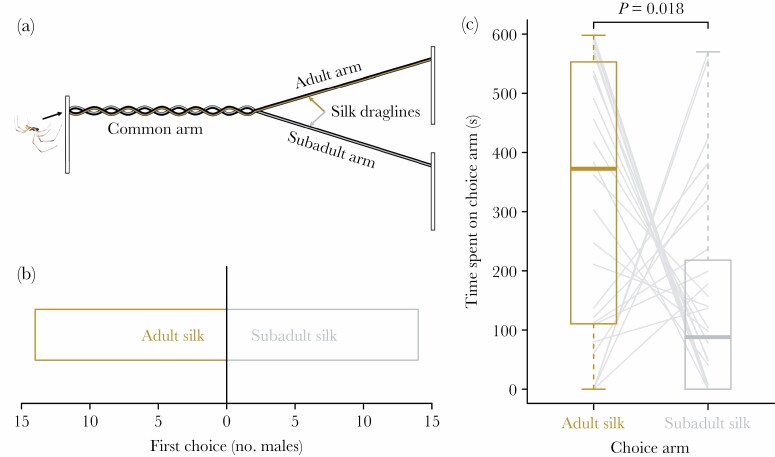
Choices of *Latrodectus geometrics* males (*n* = 30) exposed to the silk of adult and subadult females in a Y-maze. (a) Y-maze setup with a male entering at the far left. (b) Males chose the subadult and adult female arm as first equally often (binomial test). (c) Males spent more time on the adult arm than the subadult arm across 10 min trials (paired Wilcoxon signed-rank test). Boxplots illustrate medians (thick lines), interquartile ranges (boxes), and extreme values (whiskers) and pale gray lines connect the raw data for individual males.

A Y-maze choice apparatus was used to test male mate choice based on direct contact with silk (modified from the X-maze described in Scott et al. 2019; Figure 1a). The apparatus consisted of two strings, each covered in the silk of an adult or subadult female. The Y included a “common arm” with the strings intertwined to combine the cues from subadult and adult silk and two independent arms with only the silk of an adult or a subadult (Figure 1a).

To create the choice maze, each string was separately extended between two sticks and secured with alligator clips affixed to the top of each stick. To obtain silk, a female was released on one end of each string (spiders leave drag lines as they walk, Foelix 2011). Once the female walked along the whole length of the string, she was removed and placed at the beginning again. This was repeated until the female had walked three times on one string in the same direction. After this procedure, the female had secured sufficient silk on the string such that it was visible with the naked eye. When she reached the end of the string for the third time, she was gently removed with a paintbrush and returned to her cage. We randomly assigned a subadult or adult female to lay silk on the left or right string for each trial. Immediately after obtaining silk on each string, one end of each string (the end where females were first introduced) was gently unclipped, held at the unsecured tips, and gently interwoven along half of its length to create a “common arm” containing silk from both females (Figure 1a). We practiced this procedure in pilot studies and were able to prevent pulling or losing the silk during the process by minimizing the manipulation of the strings. The other halves of each string remained separate, so each contained silk from one female (choice arms: “adult arm” and “subadult arm”). The Y-shape was held in place using 3 sticks with clamps and the whole apparatus was housed in a plastic bin.

Immediately after setting up the Y-shape, a trial began when a single male was released at the beginning of the common arm so he encountered silk from both females at the same time. After the male reached the end of one of the choice arms, he could turn around and re-visit the common arm, visit the opposite choice arm, or remain on the initially chosen arm. Male behavior was observed for ten minutes and we recorded which choice arm (adult or subadult) he chose first (first choice), how many times he visited each of the choice arms (number of visits), and we used a stopwatch to record how much time males spent on each arm to the nearest second (duration). The first visit can be interpreted as a choice (e.g. Riechert and Singer 1995; Gaskett et al. 2004; Sentenská et al. 2020), particularly because in our design males were able to assess both options on the common arm (Gaskett 2007). We also measured number and total duration of visits to obtain several possible indicators of male choice (Andrade and Kasumovic 2005; Sentenská and Pekár 2019), as these variables might indicate the persistence of males in seeking the female whose presence is indicated by the silk cues.

### Statistical analysis of study 1

We ran all analyses in R (R Core Team 2020, version 4.0.0, software RStudio). We first asked whether males were more likely to visit the subadult arm as their first choice using a binomial test. We also compared the number of visits to the subadult and adult arms for each male using a paired Wilcoxon signed-rank test (data were non-normal but roughly symmetrically distributed about the median). To determine whether males in the Y-maze spent more time in contact with subadult or adult silk, we compared the total time spent by each male on the two-choice arms using a paired Wilcoxon signed-rank test (after applying a square-root transformation on these non-normal, right-skewed data to meet the assumption of symmetrical distribution about the median).

### Study 2: male courtship investment and mating success based on web and female cues

We placed adult and subadult females into empty webs made by females of the same or the opposite developmental stage (web-swap experiment), then released males into these webs to determine whether male courtship was affected by female’s silk, bodies, or both. To create experimental webs, unmated females (55 adult and 55 subadult females) were put on metal frames (11 × 8 × 8 cm) and were left to build a web. After three days the females were coaxed to the edge of their web with a paintbrush and gently removed with soft forceps so their webs were not damaged. For our experimental treatments, thirty subadult females were put into webs of thirty adult females (SA group); while thirty adult females were placed in webs of thirty subadult females (AS group). For controls, twenty-five adult females were placed into webs of twenty-five different adult females (AA group) and twenty-five subadult females were placed into webs of twenty-five different subadult females (SS group). The webs were spun by the same females that were later used in the experiments, but females were never placed back into their own web or a web spun by a female from the same family line. Subadult females were used for the experiment two to three days after the color change of their genital area was observed and were checked for molting after the experiments. Adult females were used in trials 5–13 days after the molt to maturity.

To ensure swapped females did not add additional silk to the host web, each focal female’s spinnerets were sealed with cyanoacrylate glue (LePage ultragel super glue). Females were immobilized for this procedure by placing them with ventral side facing up, on a soft pad, and then covering them with a mesh that was fixed to the pad with insect pins. The mesh had one large opening through which the posterior part of the abdomen protruded. A small drop of glue was placed on the spinnerets while the female was viewed under a dissecting microscope. The glue was left to dry for ten minutes and after that, the female was removed from the pad and put into an empty web constructed by another female as per the treatments outlined above.

Each “swapped” female was left to habituate on their new web for 10 min, by which time all females had settled and were quiescent. An unmated male was then introduced and male and female activity across the full web surface was recorded on digital video (HD-SDI cameras, Navitar macro zoom lens) for 24 h. Males were never exposed to a female or web produced by a female from their own family line. Adult males were used 5–20 days after the molt to maturity. Individuals of both sexes were used only once in this study and were not used in Study 1.

**Figure 2:**
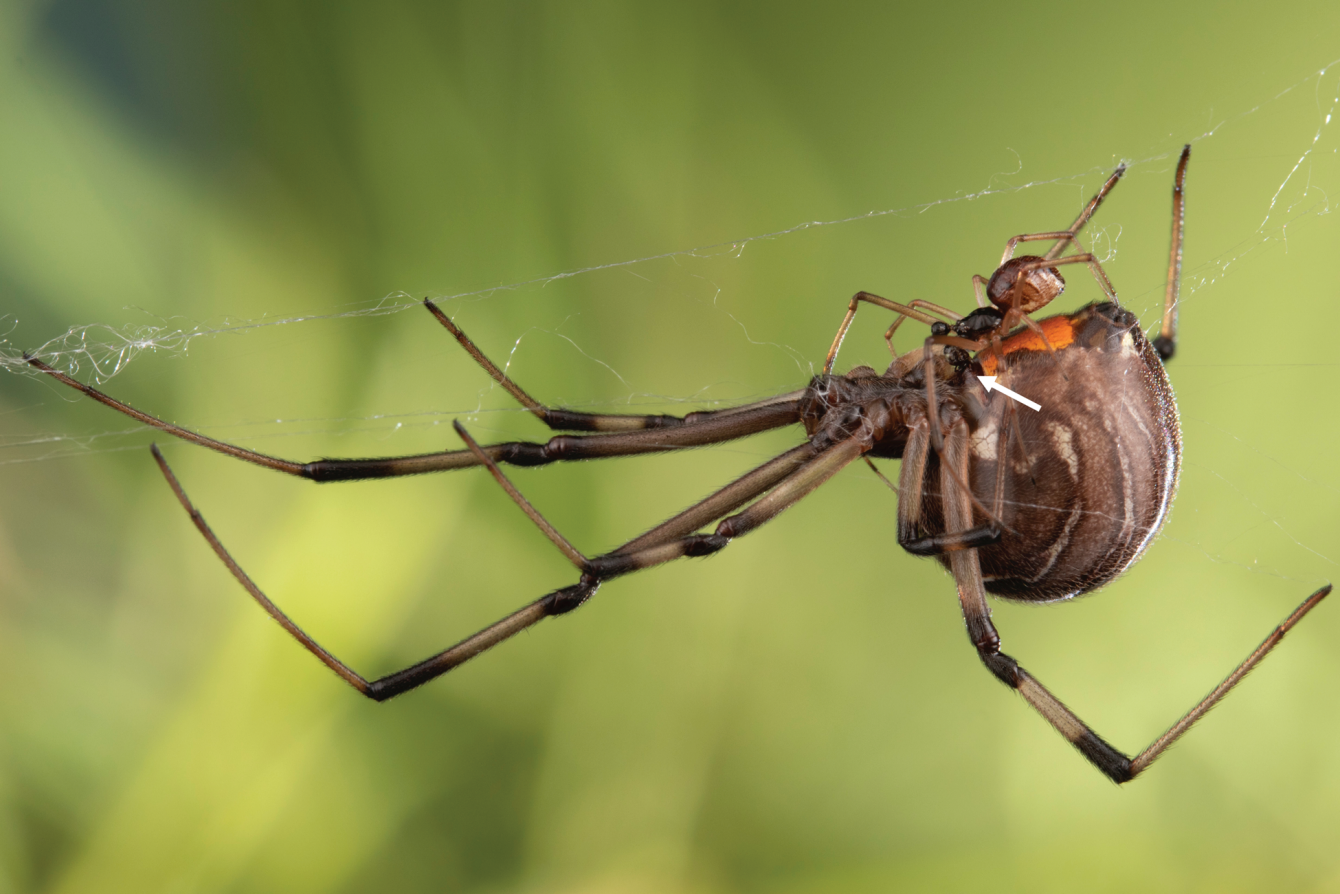
Male (smaller) and female (larger) *Latrodectus geometricus* in copulatory posture. The white arrow indicates the male’s left copulatory organ (pedipalp) making contact with the female’s genital opening (epigynum). Photo: Sean McCann.

One of us (LS) scored the videos and recorded male courtship behavior and the progression and outcome of mating. Mating can occur as soon as 30 min after the initial contact between male and female (LS, pers. observation), so, courtship behaviors were scored for the first 30 min after the introduction of the male to ensure a standardized observation period. Key courtship components for *L. geometricus* include web reduction behavior (adding silk and cutting and bundling sections of female’s web), vibrational signals, and contact courtship such as standing on female’s abdomen and laying silk strands on her body (Ross and Smith 1979; Segoli et al. 2008; Harari et al. 2009; Scott et al. 2015). We recorded the following behaviors: (1) the occurrence of silk laying (i.e. male lifts his abdomen and performs lateral movements while pulling silk out of their spinnerets with the last pair of legs), (2) the occurrence of mate binding (i.e. male lays silk in the same manner as described above, but directly on the female), (3) the latency to the first contact between male and female, which is an indication of the time the male invests before direct interactions with the female, (4) occurrence of and latency to the first mount (i.e., male climbs onto the female and adopts the copulatory posture, standing on female’s ventral abdomen with his pedipalps near the female’s genitalia), which requires female compliance as well as male attempts, (5) occurrence of and latency to the first copulation (i.e., male inserts one of his paired copulatory organs into one of paired female’s copulatory openings; Figure 2), and (6) whether males that achieved at least one copulation performed a somersault.

Although at least 25 trials were established for each treatment, final sample sizes for analyzing courtship behavior vary (SA = 12, AS = 25, AA = 22, SS = 19). Trials were excluded from analyses if the female did not stay in the web, or the view available in the recording did not allow scoring of male behavior. In one case we could not determine the age of a subadult female (in the SS group) because she died before molting, so the data for this female were also excluded. Finally, our post-hoc determination of the age of subadult females (in days before the molt to maturity) suggested that being placed on adult webs accelerated the timing of molting for females in the SA group relative to the SS group (see Results). To ensure that the pre-molt age range of subadult females in each treatment group was similar (2–5 days before moult), we, therefore, excluded from the courtship and mating behavior analyses ten SA females that molted within 1 day of being placed in the experimental treatment (see Results and Supplementary Material). It is important to standardize the time before the final molt because subadults that are very close to molting (e.g. ≤1 day) may show physiological shifts (Foelix 2011) that could affect mating outcomes (see Supplementary Material Table S3, Figure S2).

### Statistical analysis of study 2

To test the effect of the type of web (produced by a subadult or adult female) and the stage of the female (subadult or adult) on the occurrence and timing of male behaviors and mating outcomes, we ran generalized linear models (GLMs) in R version 4.0.0. In our experiments, males were between 1 and 3 weeks old. This was not expected to affect the outcome of the experiment because male age was similar across treatments. In addition, males may live up to 36 days after being field-captured as adults (data from the similarly-sized *L. hasselti*, Andrade 2003), and healthy males court females throughout their lives (MCBA, pers. observation). Our visual inspection of the data was consistent with this assumption, so we did not include male age as a covariate in our models to avoid losing power. For binary dependent variables (occurrence of silk laying, silk binding, mounting, copulation, and somersaulting), we ran logistic regression models (GLMs with binomial distribution and logit link) and for non-negative continuous dependent variables (latency to first contact with the female, latency to first mount, and latency to first copulation from the time of the first mount) we ran GLMs using the gamma distribution and log link. We began by including female stage, web type, and their interaction as predictors in all models. We ran likelihood ratio tests (using the anova function in R) to test the significance of fixed effects in each model. If the interaction term was not significant we re-ran models with only the main effects and reported results of the simplified models. We also asked whether mate binding was associated with female receptivity using a logistic regression with occurrence of mounting as the response variable and occurrence of silk binding and female stage as predictors (an interaction term was initially included and then removed from this model after confirming it did not have a significant effect). We report descriptive statistics as estimated marginal means ± SE from final models in the results.

## RESULTS

### Study 1: male mate choice based on female silk

Males did not express a preference in terms of which choice arm they visited first (binomial test: *n* = 28, *P* = 1.0; Figure 1b) and they visited the adult and subadult arms equally often as they explored the Y-maze throughout the trial (Wilcoxon signed-rank test: *V* =137, *P* = 0.97). However, males spent significantly more time (median 4.8 min more) on the adult arm than on the subadult arm (Wilcoxon signed-ranked test: *V* = 99, *P* = 0.02; Figure 1c), suggesting a preference for approaching and courting adult females based on silk-borne contact cues.

### Study 2: male courtship investment and mating success based on web and female cues

**Figure 3:**
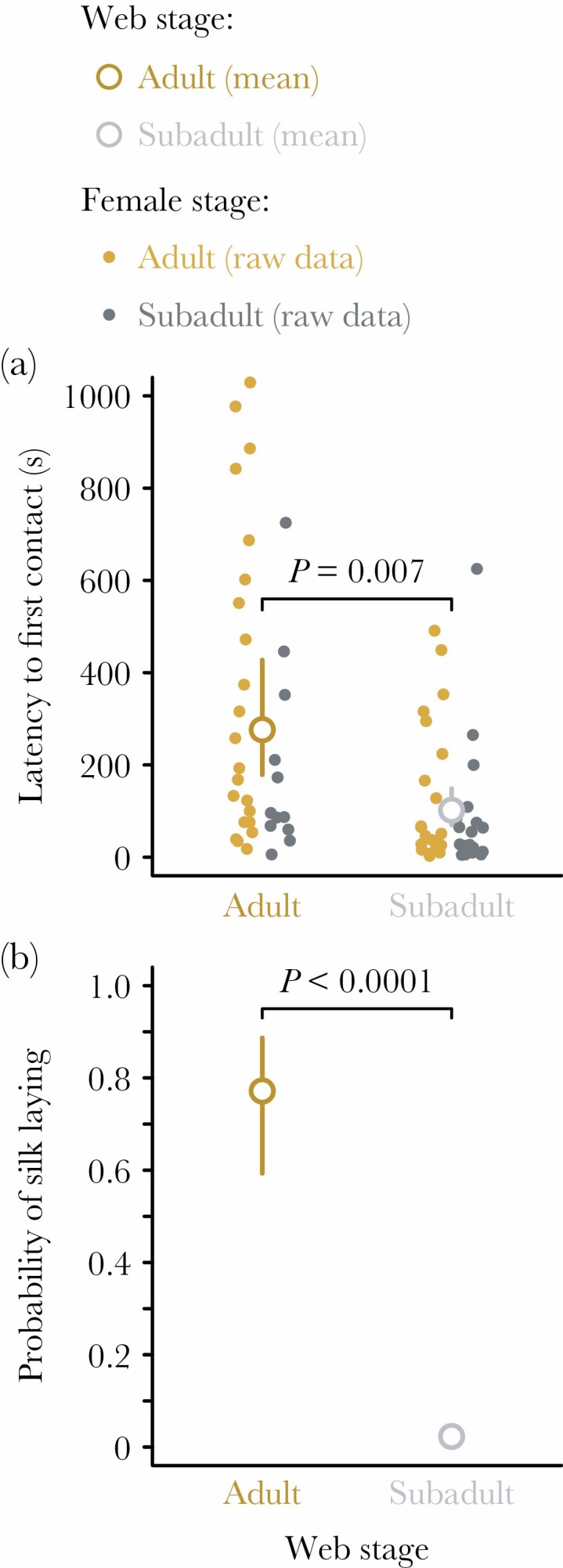
Occurrence and timing of elements of courtship and mating for *Latrodectus geometricus* males that were influenced by the status of the female that produced the web they courted on, regardless of the status of the female present in the web (subadult and adult females were staged in webs built by other subadult or adult females in a fully factorial design). (a) Males had longer latencies to first contact with females staged in adult webs than subadult webs. (b) Males were much more likely to engage in silk laying on the webs of adult females than subadult females. Large points represent estimated marginal means flanked by their 95% confidence intervals from GLMs (back-transformed from the logit or log scale) and small circles represent raw data.

**Figure 4:**
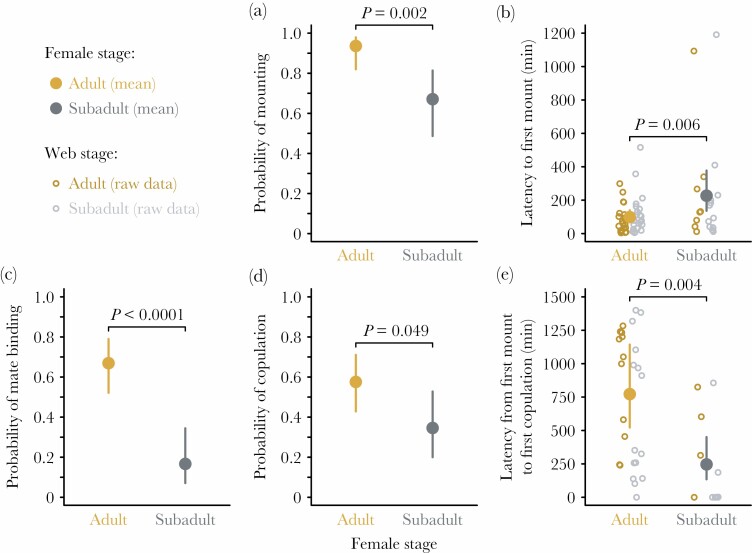
Occurrence and timing of elements of courtship and mating for *Latrodectus geometricus* males that were influenced by the status of the female they courted, regardless of the status of the female who built the web they courted on (subadult and adult females were staged in webs built by other subadult or adult females in a fully factorial design). (a) Males were more likely to mount adult females than subadult females. (b) Males mounted adult females sooner than subadult females. (c) Males were more likely to engage in mate binding when courting adult than subadult females. (d) Males were more likely to copulate at least once with adult females than subadult females. (e) The latency to copulation (from the first mount) was greater for adult females than subadult females.

Some male behaviors were strongly influenced by the stage of the female who built the web on which they courted (Figure 3; Supplementary Material Table S1), whereas others were influenced by the stage of the female inhabiting the web (Figure 4; Supplementary Material Table S1). Males contacted females earlier when courting on subadult webs (102 ± 20 s after the beginning of the trial) compared with adult webs (276 ± 61 s after the beginning of the trial), regardless of the stage of the female they were courting (LR χ ^2^ = 11.6; *P* = 0.0007; Figure 3a), indicating that males invest less time in courtship on the webs of subadult females before approaching the resident female. The probability of successfully mounting was greater for males courting adult females (0.94 ± 0.04) compared with subadult females (0.67 ± 0.09) regardless of which the web type (LR χ ^2^ = 9.2; *P* = 0.002; Figure 4a), and mounting also occurred earlier with adult females (after 99 ± 18 min) than with subadults (after 227 ± 59 min), regardless of web type (LR χ ^2^ = 7.6; *P* = 0.006; Figure 4b).

Males were much more likely to lay silk on the webs of adults (0.77 ± 0.08) than of subadults (0.02 ± 0.02), regardless of the stage of the resident female (LR χ ^2^ = 53.9; *P* < 0.0001; Figure 3b). In contrast, the probability of mate binding was greater for males courting adult females (0.67 ± 0.07) than subadult females (0.17 ± 0.07), regardless of web type (LR χ ^2^ = 19.5; *P* < 0.0001; Figure 4c). Moreover, males that engaged in mate binding were more likely to successfully mount females (adopting the copulatory posture; Figure 2, probability of mounting was 0.97 ± 0.03) than were males that did not engage in binding (probability: 0.71 ± 0.07; LR χ ^2^ = 4.8; *P* = 0.03; Supplementary Table S2). Females can prevent males from mounting by moving or kicking, and mating occurs only while males are in this posture, so reaching this stage of courtship implies female receptivity. There was no interactive effect of mate binding and female stage on mounting, but consistent with the higher probability of mounting adult females reported above, there was also a marginal effect of female status on mounting in this model (LR χ ^2^ = 2.9; *P* = 0.09).

Consistent with the results for mounting, the probability of at least one successful copulation was greater for males courting adult females (0.58 ± 0.07) than subadult females (0.35 ± 0.09), regardless of which type of web they were on (LR χ ^2^ = 3.9; *P* = 0.049; Figure 4d). The latency from the first mount to the first copulation (if any) was also much greater for adult (773 ± 154 min) than subadult females (246 ± 76 min) regardless of web type, indicating that males invest much more time on proximal courtship with adults (LR χ ^2^ = 8.2; *P* = 0.004; Figure 4e). Moreover, for males that copulated at least once, the probability of somersaulting was much greater when mating with adult females (0.96 ± 0.04) than with subadults (0.09 ± 0.09), regardless of web type (LR χ ^2^ = 29.7; *P* < 0.0001; Table 1).

**Table 1:** Summary of mating outcomes for male *Latrodectus geometricus* males assigned to females in each treatment group. Two copulations (one into each of the female’s paired copulatory openings) are required for a complete mating, and males typically perform a “somersault”, placing their abdomen near the female’s mouthparts, during each copulation with unmated adult females

Female stage	Web stage	*n* ^a^	Copulation no.		Mated	Somersault no.		Somersaulted^b^
			1	≥2		1	≥2	
Adult	Adult	21	3	8	52.4%	3	8	100.0%
	Subadult	24	5	10	62.5%	6	8	93.3%
Subadult	Adult	12	1	3	33.3%	0	0	0.0%
	Subadult	19	5	2	36.8%	1	0	14.3%

^a^Pairs for whom insertion could be unambiguously determined.

^b^Percentage of males that mated.

### Early maturation of subadult females transplanted onto adult females’ webs

Despite subadult females being randomly assigned to adult or subadult webs for the web-swap experiment, our post-hoc determination of female age on the day of mating trials revealed significant differences between the two treatment groups (Wilcoxon signed-rank test; *W* = 231.5, *P* = 0.002; Supplementary Material Figure S1). Subadults placed on adult webs matured 20% sooner (median 2 days after trials; range 0–5 days) than subadults placed on the webs of other subadults (median 3 days after trials; range 2–5 days), suggesting an effect of contact with adult female silk-bound sex pheromone on the timing of molting. We removed these females from our analyses to ensure that the age of females (number of days before the molt) was consistent across our treatments, and thus would not lead to spurious differences in courtship behavior or mating outcomes.

## Discussion

We investigated male mate choice in *Latrodectus geometricus* spiders using two experiments that examined male responses to contact with female silk in the presence and absence of females at two different developmental stages (adult or subadult). Mating with adult or subadult females has very different implications for male energy expenditure and post-mating survival in this species (Biaggio et al. 2016). Here we show that males are clearly able to perceive subadults based on silk cues, as they were equally likely to explore subadult compared with adult dragline silk in our Y-maze. However, males spent significantly more time on draglines of adult females, which confirms previously observed male preference for adult over subadult females based on air-borne cues (Waner et al. 2018; Sentenská et al. 2020). Further, in accordance with previous studies of *L. geometricus* (Biaggio et al. 2016; Sentenská et al. 2020), we observed males to perform different courtship towards adult and subadult females, with our design allowing a more precise dissection of the cues that trigger this effect. We observed that some behaviors are driven by contact with the web (silk laying) whereas others were driven by contact with the female (mate binding and somersaulting). Typically, only contact with an adult female or her web triggered these behaviors suggesting that males invest less into mating with subadult females. Additionally, adult females were mounted more quickly and with a higher probability than subadults, suggesting that subadult females are less receptive to mating attempts, despite being physically capable of mating.

Previous studies showed that males approach adult females more often than subadults when simultaneously exposed to their air-borne pheromones (Waner et al. 2018; Sentenská et al. 2020). In this study, we exposed males to silk-borne pheromones in a Y-maze arena in which the first visit to an experimental arm (subadult or adult silk only) may indicate a male preference (e.g. Gaskett et al. 2004). However, in our experiment, neither males’ first visits nor their visit frequency were affected by female developmental stage, indicating no initial preference based on silk-borne cues. However, males did spend significantly more time on adult silk, suggesting that when given a simultaneous choice between cues produced by subadult and adult females, they prefer to approach and court the adult.

The apparent advantages of immature mating, including reduced courtship and lack of cannibalism, may more accurately represent evidence for higher male investment into adult females. For example, sexual cannibalism is triggered by male behavior in *L. geometricus* and typically occurs only after the male somersaults onto the female’s mouthparts (Segoli et al. 2008). In the congener *L. hasselti* this “self-sacrifice” is considered an adaptive male behavior, as it prolongs the copulation and decreases female re-mating propensity (Andrade 1996). In a non-sacrificial congener, *L. tredecimguttatus*, cannibalism also relates to prolonged copulation (Golobinek et al. 2021). In *L. geometricus*, the rate at which males somersault with adult females is related to female reproductive value; almost 80% of males somersault when copulating with unmated females but only about 30% of males offer themselves to previously mated females (Segoli et al. 2008). Here we found that variation in male somersault behavior was related to female body cues as males typically somersaulted with adult females but did so only rarely with subadults. Therefore, the absence of cannibalism can be interpreted as the male’s rather than the female’s decision. We also found that for males who successfully mounted a female, the subsequent latency to copulation was shorter with subadults. This reflects less time spent courting by the male on and around the female’s body before copulation. The reduced courtship observed in this and other studies of immature mating in *Latrodectus* spp. (Biaggio et al. 2016; Baruffaldi and Andrade 2017; Sentenská et al. 2020) may be best understood as resulting from males’ lower investment into subadult females rather than as an advantage males can gain from mating with subadults. Nonetheless, it is difficult to distinguish proximate from ultimate causation, namely whether males have evolved to invest less in subadult females because these females are less likely to mate, or whether female reluctance is caused by lower investment from the male side. Future studies that seek to disentangle these complexities would be valuable.

It is not immediately obvious why males would invest less into mating subadults than adults because these females have similar fecundity and fertility (Biaggio et al. 2016). Mechanistically, this may arise from lower production of pheromones at the subadult stage. In terms of fitness effects for males, adult females are ready to produce egg sacs, whereas subadults still have to undergo the final molt with attendant mortality risk and time delay (ones 1941; Horner and Starks 1972; Tanaka 1984), which may offset any benefits of mating with subadults. Additionally, although not detected in a previous study (Sentenská et al. 2020, but see Biaggio et al. 2016), in this experiment we observed that subadult females mated less often than adult females, even after male mounting. This suggests subadults are less receptive to male mating attempts, possibly contributing to male preference for adult females. Previous studies showed that *L. geometricus* males achieved matings faster with immature females (Biaggio et al. 2016; Waner et al. 2018). On the contrary, in our experiments, the latency to mount subadult females was significantly greater compared with adult females and fewer males achieved mounting with subadults. These results likely do not reflect the male’s courtship effort but rather the female’s receptivity to mating, because males approached females repeatedly during trials and we observed subadult females moving away or kicking the male (although we did not quantify these deterrent behaviors). In the congener *L. hasselti,* however, subadult females showed similar deterrent behaviors towards males’ mounting attempts, and also showed longer latency to copulation than in matings with adult females, despite the reduced courtship (Baruffaldi and Andrade 2017). Therefore, although able to mate, subadult females seem to be more reluctant to do so than adult females. Female willingness to mate seems to be affected by the age of subadult females—across all subadult females, the probability of copulating increases as females approach the molt to maturity (Supplementary Material Figure S2), with the subadult females within one day of maturity copulating at similar rates to adults.

Unlike other courtship components, web reduction (accompanied by silk laying) is apparently triggered solely by the contact sex pheromone on the adult female’s silk (Figure 3b). In other studies, web reduction appears to decrease adult female aggression toward males and increase female quiescence (Scott et al. 2012; DiRienzo et al. 2019), suggesting this behavior may affect female receptivity. We found no evidence for such effects in subadults that experienced web reduction. One of the key roles of web reduction is to alter air-borne signals released from the webs of adults (Watson 1986; Gaskett 2007; Scott et al. 2015). It seems likely that males forgo reduction of subadult webs because subadult *L. geometricus* females are not highly attractive to rival males (Waner et al. 2018; Sentenská et al. 2020; this study). In contrast to web reduction, mate binding occurred more frequently with adults, and thus was apparently not influenced by the origin of the web itself. Mate binding may increase or accelerate female receptivity to mate (Scott et al. 2018), perhaps because it allows chemical validation of male identity by the female (Ross and Smith 1979). In our experiment, this courtship component was positively associated with the occurrence of mounting, and thus likely with female receptivity. Notably, 100% of males that bound subadults with silk successfully mounted, compared with only 61.5% who did not bind females. This association between higher courtship investment (in the form of mate binding) and increased subadult mating frequency is consistent with the idea that males choose to invest less in courtship with subadults, despite their ability to mate.

One intriguing outcome of the design of our web-swap experiment was an apparent acceleration of maturation of subadult females that contact the silk of adult females, which may suggest that female development may respond to social cues of intrasexual competition or facilitation (e.g., see Kasumovic and Brooks 2011; Neumann and Schneider 2016; Quiñones-Lebrón et al. 2021). This has been demonstrated in locusts (both sexes, *Schistocerca gregaria*, Mahamat et al. 1993), and in male *Latrodectus* spiders (which respond to both male and female pheromones, Kasumovic and Andrade 2006). However, although females of some spiders are attracted to (e.g. Roland 1983; Trabalon and Assi-Bessekon 2008; Krafft and Cookson 2012) or repulsed by (Clark and Jackson 1994) the silk of other females, developmental acceleration has not previously been indicated for female spiders, to our knowledge. In some moth species, females exposed to signals of other females initiate and intensify chemical signaling at lower age, likely to increase the probability of successful mating in face of competition for available males (Rehermann et al. 2016) or to increase the attraction of males through joint signaling (Lim and Greenfield 2007). The encounter rate between the sexes in widow spiders is low and males experience high mortality (>80%) during mate search (Andrade 2003; Segev et al. 2003; Segoli et al. 2006; Scott et al. 2019) and this in addition to sexual cannibalism may lead to a shortage of males. Although more rigorous testing is required, we speculate that subadult females of *L. geometricus* that mature more quickly in the presence of rival females may reduce the risk of delays to mating that would result from losing in competition for mates with already-adult females. Testing of this idea would require assessing patterns of development, mate attraction, and reproductive fitness for females in nature. If intrasexual competition does in fact favor adaptive plasticity in females through accelerated development, it suggests a previously unanticipated, strong effect of sexual selection on females that would shift with female phenology in nature.

We conclude that brown widow males prefer to approach and court adult females over subadult females based on both contact and air-borne cues, and that they invest more into costly courtship behavior, including self-sacrifice, with adult females. This preference may be driven by the greater likelihood of mating success with adults and their readiness for oviposition, which likely outweigh the apparent energy and fitness costs of a long courtship ending with cannibalism. These results highlight the importance of considering the context in which mating interactions occur for understanding how sexual selection operates in nature. At first glance cannibalism clearly carries a high fitness cost, but in a system where mate search is extremely risky and the likelihood of encountering multiple females is low, high investment into cannibalistic females can be favored.

## Supplementary Material

arac023_suppl_Supplementary_Material

## Data Availability

Analyses reported in this article can be reproduced using the data and codes provided by Scott et al. (2022).
