# Supplementary material for: Risky business: males choose more receptive adults over safer subadults in a cannibalistic spider
Source: Behav Ecol. 2022 Apr 25;33(4):688–97. doi: 10.1093/beheco/arac023 (PMC9262164; doi:10.1093/beheco/arac023)
Supplement: arac023_suppl_Supplementary_Material [file arac023_suppl_supplementary_material.docx]

**Supplementary Material for Risky business: Males choose more receptive adults over safer subadults in a cannibalistic spider**

Lenka Sentenská^1,2^, Catherine Scott^1^, Pierick Mouginot^3^, Maydianne C.B. Andrade^1^

^1^ Department of Biological Sciences, University of Toronto Scarborough, 1265 Military Trail, Scarborough, Ontario, Canada M1C 1A4

^2^ Department of General and Systematic Zoology, University of Greifswald, Loitzer Strasse 26, 17489 Greifswald, Germany

^3^ PSL Université Paris: EPHE-UPVD-CNRS, USR 3278 CRIOBE, Université de Perpignan, 52 Avenue Paul Alduy, CEDEX 9, 66860 Perpignan, France

**Table S1.** Results of generalized linear models assessing the effects of type of web (subadult or adult) and female stage (subadult or adult), on the latency to (gamma distribution and log link) or occurrence of (binomial distribution and logit link) elements of courtship and mating behaviour by *Latrodectus geometricus* males.

| Response variable | Model coefficient  (reference level) | Estimate | SE | *t*/*Z** | *P* |
| --- | --- | --- | --- | --- | --- |
| latency to first contact | intercept | 5.83 | 0.24 | 24.13 | <0.0001 |
|  | female stage (adult) | -0.41 | 0.30 | -1.40 | 0.17 |
|  | web stage (adult) | -1.00 | 0.29 | -3.43 | 0.001 |
| mount occurrence | intercept | 2.53 | 0.67 | 3.78 | 0.0002 |
|  | female stage (adult) | -1.98 | 0.72 | -2.76 | 0.006 |
|  | web stage (adult) | 0.31 | 0.66 | 0.48 | 0.63 |
| latency to first mount | intercept | 4.54 | 0.24 | 18.92 | <0.0001 |
|  | female stage (adult) | 0.83 | 0.31 | 2.67 | 0.01 |
|  | web stage (adult) | 0.09 | 0.30 | 0.32 | 0.75 |
| copulation occurrence | intercept | 0.15 | 0.39 | 0.37 | 0.71 |
|  | female stage (adult) | -0.94 | 0.49 | -1.94 | 0.05 |
|  | web stage (adult) | 0.32 | 0.48 | 0.66 | 0.51 |
| latency from first mount to copulation | intercept | 6.92 | 0.28 | 24.87 | <0.0001 |
|  | female stage (adult) | -1.14 | 0.36 | -3.14 | 0.003 |
|  | web stage (adult) | -0.54 | 0.34 | -1.60 | 0.12 |
| silk laying  occurrence | intercept | 1.10 | 0.48 | 2.29 | 0.02 |
|  | female stage (adult) | 0.24 | 0.80 | 0.31 | 0.76 |
|  | web stage (adult) | -4.97 | 1.10 | -4.53 | <0.0001 |
| mate binding occurrence | intercept | 1.06 | 0.44 | 2.39 | 0.02 |
|  | female stage (adult) | -2.31 | 0.59 | -3.95 | <0.0001 |
|  | web stage (adult) | -0.71 | 0.53 | -1.33 | 0.18 |
| somersault occurrence | intercept | 3.30 | 1.37 | 2.41 | 0.02 |
|  | female stage (adult) | -5.52 | 1.46 | -3.77 | 0.0002 |
|  | web stage (adult) | -0.13 | 1.50 | -0.09 | 0.93 |

**t*-statistics or *Z*-statistics are reported for models with latency or occurrence response variables, respectively

**Table S2.** Results of a generalized linear model (using binomial distribution and logit link) assessing the effect of mate binding (yes or no) on the occurrence of mounting (yes or no) by *Latrodectus geometricus* males during courtship trials.

| Response variable | Model coefficient  (reference level) | Estimate | SE | *Z* | *P* |
| --- | --- | --- | --- | --- | --- |
| mount occurrence | intercept | 1.76 | 0.67 | 2.62 | 0.008 |
|  | mate binding (no) | 2.64 | 1.07 | 2.47 | 0.07 |
|  | female stage | -1.24 | 0.76 | -1.62 | 0.11 |

**Additional Analyses**

We found that the median number of days prior to sexual maturity for subadult females (‘age’) differed depending on what web type they were staged in (discussed in the main text). Despite random allocation to treatments, only females staged on adult webs were 0 or 1 day prior to maturity (determined post-hoc) on the day of mating trials (Fig. 1).

We hypothesized that females within 1 day of their final moult might behave differently than other immature females due to the physiological changes of the approaching moult (e.g., Foelix 2011 as cited in the body of the paper). To determine whether there is a relationship between the age of subadult females (# days prior to the moult to maturity) and the occurrence of at least one copulation during mating trials, we ran a logistic regression with female age as a predictor and the occurrence of at least one copulation as the response variable, using pooled data for all subadult females (whether they were staged in the web of another subadult or an adult female). There was a significant association between female age and copulation, with females closer to the moult (particularly between 0 and 1 day before moulting) being more likely to copulate (Table S1, Fig. S2).


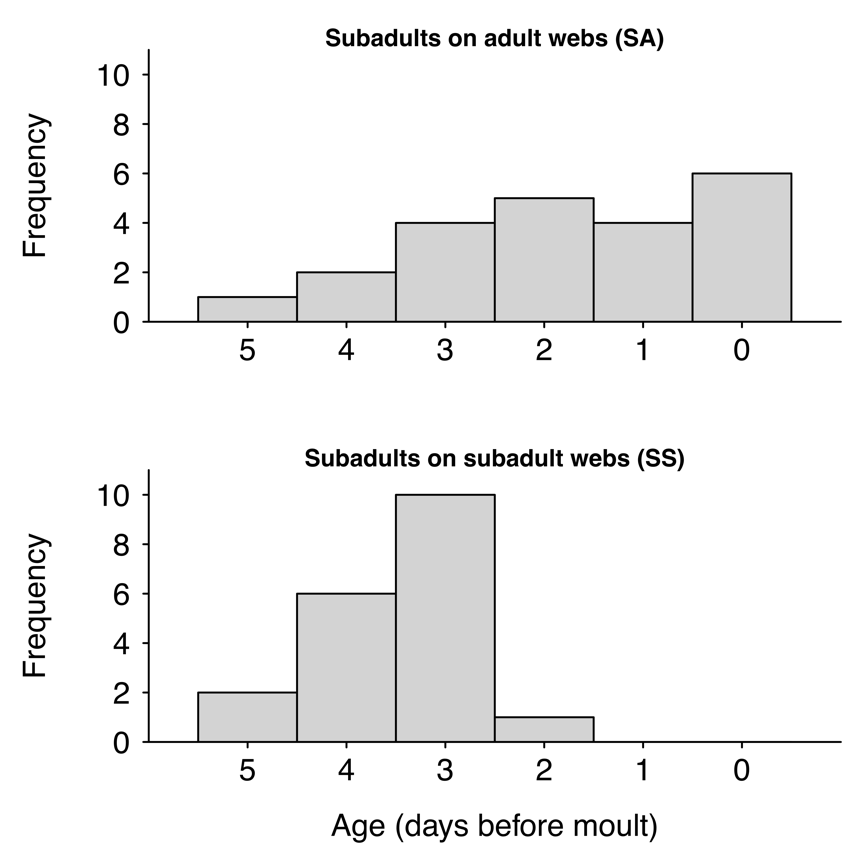


**Figure S1.** Histograms of the age distributions of subadult *Latrodectus geometricus* females staged on the webs of adult females (n = 22) or adult females (n = 20) on the day of mating trials.

**Table S3.** Results of a logistic regression to determine the effect of female age on the occurrence of mating by subadult *Latrodectus geometricus* females.

|  | Estimate | SE | *Z* | *P* |
| --- | --- | --- | --- | --- |
| Intercept | 1.56 | 0.83 | 1.88 | 0.061 |
| Female age | 0.64 | 0.28 | 2.30 | 0.021 |


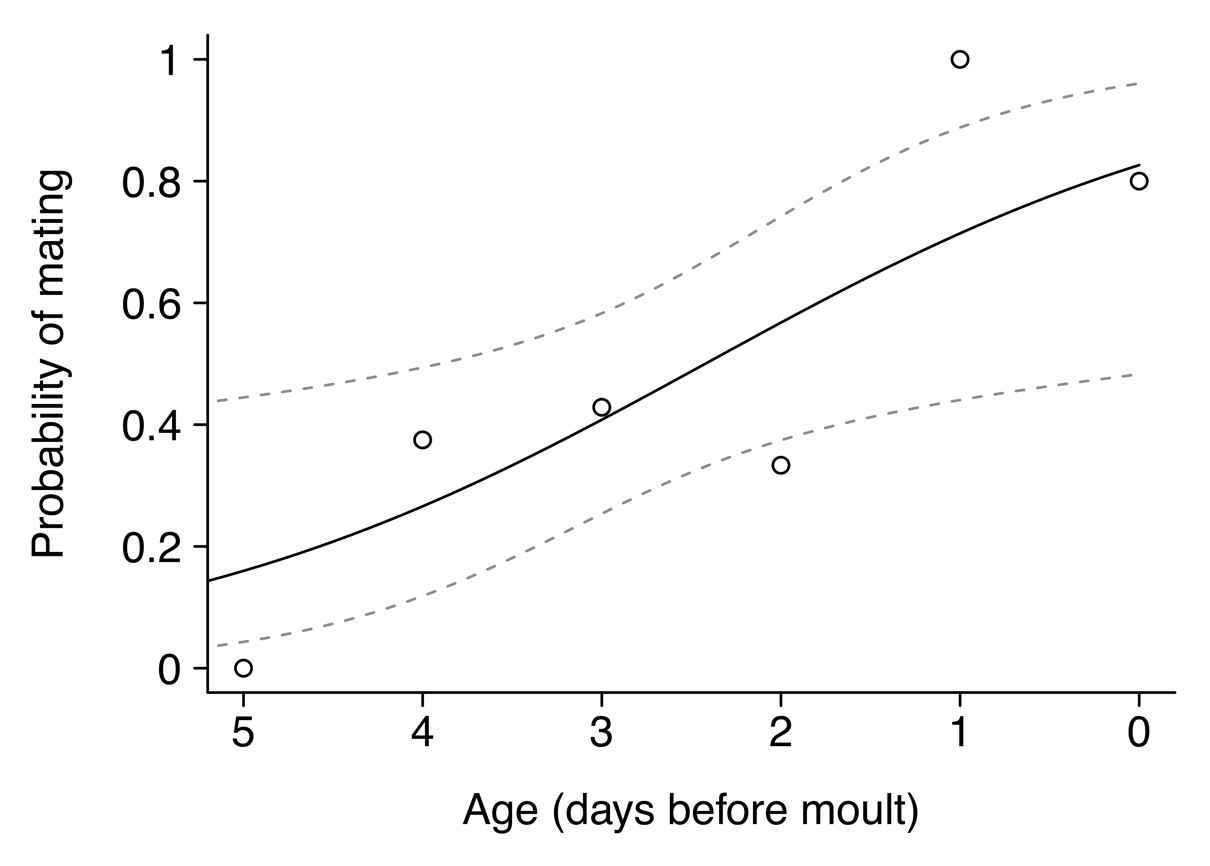


**Figure S2.** Relationship between the age of subadult *Latrodectus geometricus* females and mating (at least one copulation) in mating trials in which females were staged in the webs of adult females (n = 22) or different subadult females (n = 20). Points display the raw data, the solid line is the predicted fit from a logistic regression (back-transformed from the logit scale) and the dashed grey lines represent an approximate 95% confidence interval.

When subadult females 0 or 1 days prior to the moult to maturity were excluded from the dataset, there was no relationship between female age and probability of mating (Table S2). We used the reduced dataset without these females for all analyses reported in the main text, so that the results would not be influenced by the higher propensity of mating by females closer to moult to maturity, who only occurred in the SA group.

**Table S4.** Results of a logistic regression to determine the effect of female age on the occurrence of mating by subadult *Latrodectus geometricus* females for the subset of females that were 2–5 days prior to the moult to maturity.

|  | Estimate | SE | *Z* | *P* |
| --- | --- | --- | --- | --- |
| Intercept | 0.52 | 1.46 | 0.35 | 0.72 |
| Female age | 0.35 | 0.44 | 0.78 | 0.43 |
